# Supplementary material for: Radiomics-Based Machine Learning for Predicting the Injury Time of Rib Fractures in Gemstone Spectral Imaging Scans
Source: Bioengineering (Basel). 2022 Dec 21;10(1):8. doi: 10.3390/bioengineering10010008 (PMC9855073; doi:10.3390/bioengineering10010008)
Supplement: Supplementary file 1 [file bioengineering-10-00008-s001.zip › bioengineering-2015803-supplementary.pdf]

Random Forest

Table S1. Random Forest-Performances of the radiomics-based model for training and testing set in predicting rib fracture at 30 days.

Fracture time <30 days	Training set (70%)		
	Precision	Recall	F1-score
RF model(overfitting)			
positive	1.0	1.0	1.0
Negative	1.0	1.0	1.0
Macro avg	1.0	1.0	1.0
Weighted avg	1.0	1.0	1.0
accuracy		1.0	
AUC		1.0	
		Testing set (30%)	
positive	1.0	0.92	0.96
Negative	0.94	1.0	0.97
Macro avg	0.97	0.96	0.97
Weighted avg	0.97	0.97	0.97
accuracy		0.97	
AUC		0.99	

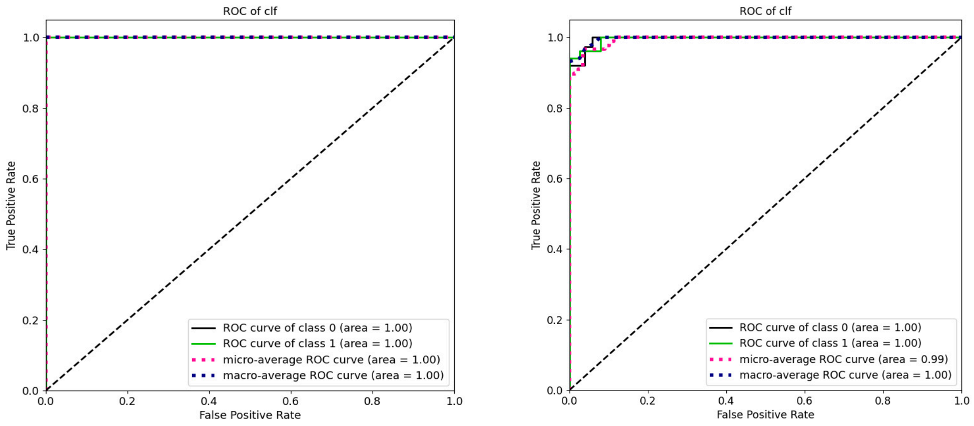

Figure S1. Random Forest-Receiver operating characteristic (ROC) curves for the training and testing set in predicting rib fracture at 30 days.

Table S2. Random Forest- Performances of the radiomics-based model for training and testing set in predicting rib fracture at 90 days

Fracture time <90 days	Training set (70%)		
	Precision	Recall	F1-score
RF model(overfitting)			
positive	1.0	1.0	1.0
Negative	1.0	1.0	1.0
Macro avg	1.0	1.0	1.0
Weighted avg	1.0	1.0	1.0
accuracy		1.0	
AUC		1.0	
		Testing set (30%)	
positive	0.93	0.94	0.93
Negative	0.95	0.93	0.94
Macro avg	0.94	0.94	0.94
Weighted avg	0.94	0.94	0.94
accuracy		0.94	
AUC		0.99	

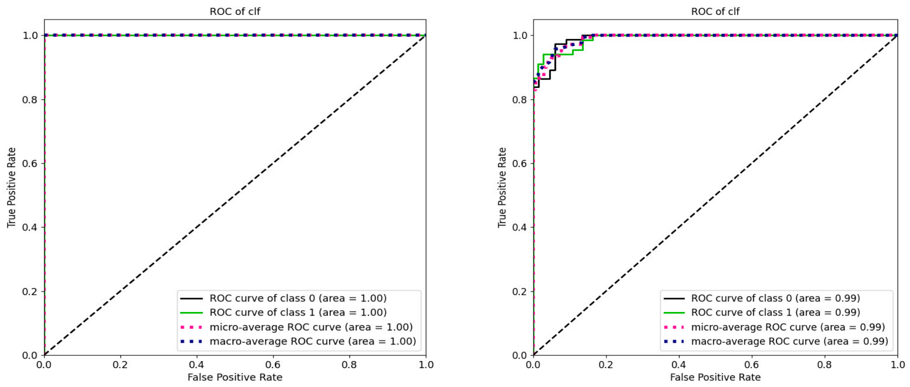

Figure S2. Random Forest-Receiver operating characteristic (ROC) curves for the training and testing set in predicting rib fracture at 90 days.

Logistic Regression

Table S3. Logistic Regression-Performances of the radiomics-based model for training and testing set in predicting rib fracture at 30 days.

Fracture time <30 days	Training set (70%)		
	Precision	Recall	F1-score
LR model(overfitting)			
positive	0.90	0.87	0.89
Negative	0.90	0.93	0.91
Macro avg	0.90	0.90	0.90
Weighted avg	0.90	0.90	0.90
accuracy		0.90	
AUC		0.96	
		Testing set (30%)	
positive	0.67	0.65	0.66
Negative	0.75	0.77	0.76
Macro avg	0.97	0.97	0.97
Weighted avg	0.97	0.97	0.97
accuracy		0.72	
AUC		0.73	

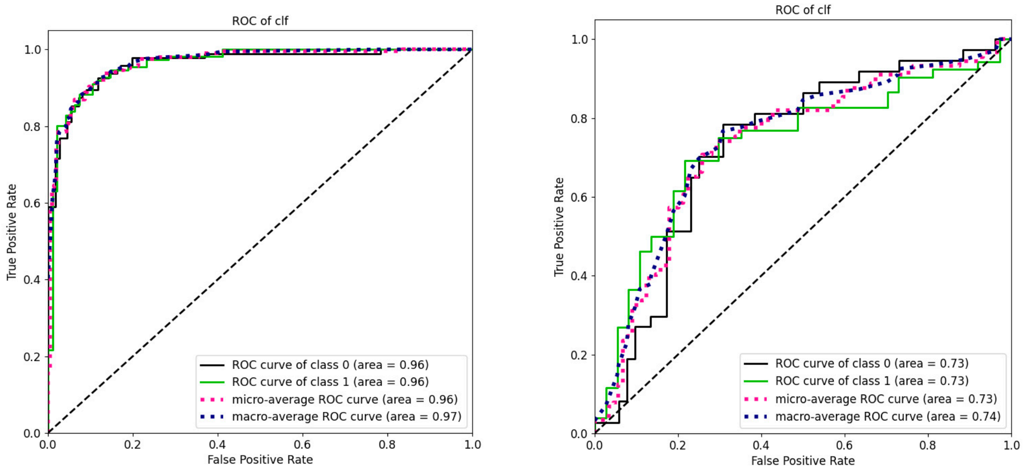

Figure S3. Logistic Regression-Receiver operating characteristic (ROC) curves for the training and testing set in predicting rib fracture at 30 days.

Table S4. Logistic Regression-Performances of the radiomics-based model for training and testing set in predicting rib fracture at 90 days.

Fracture time <90 days	Training set (70%)		
	Precision	Recall	F1-score
LR model(overfitting)			
positive	0.95	1.00	0.97
Negative	1.00	0.80	0.89
Macro avg	0.97	0.99	0.93
Weighted avg	0.99	0.99	0.99
accuracy		0.99	
AUC		0.99	
		Testing set (30%)	
positive	0.87	0.91	0.89
Negative	0.42	0.33	0.37
Macro avg	0.64	0.62	0.63
Weighted avg	0.79	0.81	0.80
accuracy		0.81	
AUC		0.85	

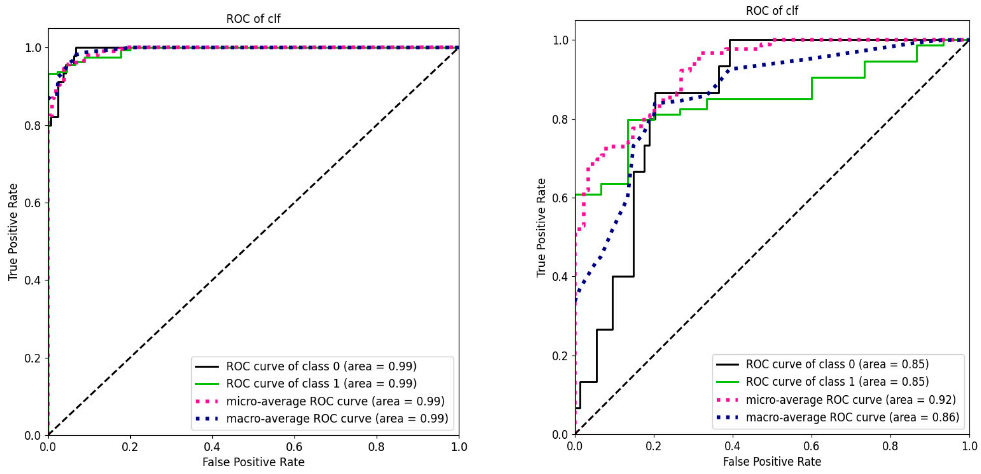

Figure S4. Logistic Regression- Receiver operating characteristic (ROC) curves for the training and testing set in predicting rib fracture at 90 days.

GaussianNB

Table S5. GaussianNB-Performances of the radiomics-based model for training and testing set in predicting rib fracture at 30 days.

Fracture time <30 days	Training set (70%)		
	Precision	Recall	F1-score
GNB model			
positive	0.81	0.88	0.84
Negative	0.89	0.82	0.86
Macro avg	0.85	0.85	0.85
Weighted avg	0.85	0.85	0.85
accuracy		0.85	
AUC		0.96	
		Test set (30%)	
positive	0.70	0.74	0.72
Negative	0.80	0.76	0.78
Macro avg	0.75	0.75	0.75
Weighted avg	0.75	0.75	0.75
accuracy		0.75	
AUC		0.80	

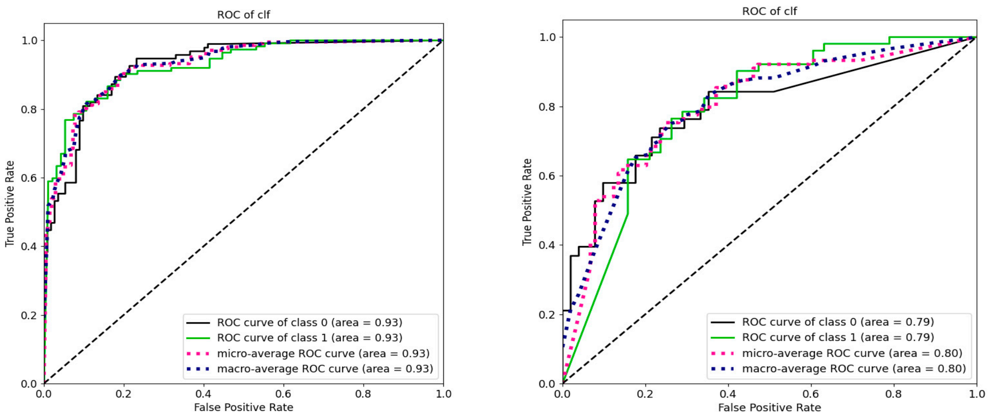

Figure S5. GaussianNB-Receiver operating characteristic (ROC) curves for the training and testing set in predicting rib fracture at 30 days.

Table S6. GaussianNB-Performances of the radiomics-based model for training and testing set in predicting rib fracture at 90 days.

Fracture time <90 days	Training set (70%)		
	Precision	Recall	F1-score
GNB model			
positive	0.97	0.84	0.90
Negative	0.61	0.91	0.73
Macro avg	0.79	0.87	0.82
Weighted avg	0.89	0.85	0.86
accuracy		0.85	
AUC		0.95	
		Test set (30%)	
positive	0.88	0.81	0.85
Negative	0.33	0.47	0.39
Macro avg	0.61	0.64	0.62
Weighted avg	0.79	0.75	0.77
accuracy		0.75	
AUC		0.76	

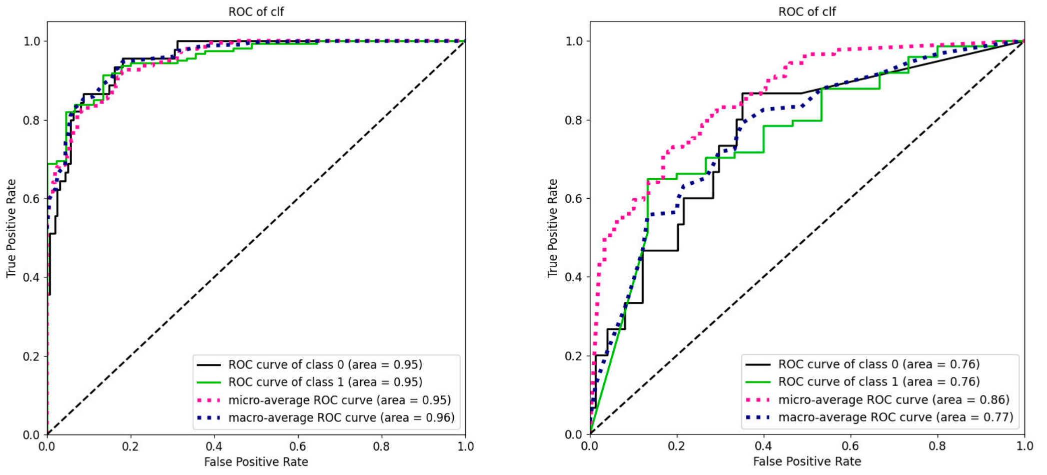

Figure S6. GaussianNB-Receiver operating characteristic (ROC) curves for the training and testing set in predicting rib fracture at 90 days.
